# Comparison of RTP dose distributions in heterogeneous phantoms with the beam Monte Carlo simulation system

**DOI:** 10.1120/jacmp.v2i1.2623

**Published:** 2001-01-01

**Authors:** Moyed Miften, Mark Wiesmeyer, Ajay Kapur, C.‐M. Charlie Ma

**Affiliations:** ^1^ Department of Radiation Oncology Duke University Medical Center Durham North Carolina 27710; ^2^ Computerized Medical Systems, Inc. St. Louis Missouri 63132; ^3^ Department of Radiation Oncology Stanford University School of Medicine Stanford California 94305; ^4^ Present address: GE Corporate Research and Development Clifton Park NY 12065

**Keywords:** dose, heterogeneous, beam, superposition, Clarkson

## Abstract

Therapeutic treatment plan evaluation is often based on examining the radiotherapy treatment planning (RTP) system dose distributions in the target and surrounding normal structures. To study the effects of tissue inhomogeneities on photon dose distributions, we compared FOCUS RTP system dose distributions from the measurement‐based Clarkson and model‐based MultiGrid Superposition (MGS) algorithms with those from the beam Monte Carlo code system in a set of heterogeneous phantoms. The phantom inhomogeneities mimic relevant clinical treatment sites, which include lung slab, lung‐bone slab, bone‐lung slab, mediastinum, and tumor geometries. The benchmark comparisons were performed in lung densities of 0.20 and $0.31g/{\text{cm}}^{3}$, and a bone density of $2.40g/{\text{cm}}^{3}$ for $5\times 5{\text{cm}}^{2}$ and $10\times 10{\text{cm}}^{2},6-$ and 15‐MV photon beams. Benchmark comparison results show that the MGS model and beam doses match better than 3% or 3 mm, and the MGS model is more accurate than the Clarkson model in all phantoms. The MGS model, unlike the Clarkson model, predicts the build‐down and build‐up of dose near tissue interfaces and penumbra broadening in lung associated with high energy beams. The Clarkson model overestimates the dose in lung by a maximum of 10% compared to beam. Dose comparisons suggest turning‐off the effective path length inhomogeneity correction in the Clarkson model for lung treatments.

PACS number(s): 87.53.–j, 87.53.Bn

## INTRODUCTION

External beam radiotherapy treatment planning (RTP) is a complex process, which involves the use of information from CT and/or MRI examinations in order to localize the target volume and surrounding normal structures. One then determines the treatment technique and beam setup, performs the dose calculation, evaluates and optimizes the plan, and verifies the plan on the simulator and treatment machine. Dose calculation and treatment plan evaluation depend strongly on the accuracy of the dose calculation algorithm in the RTP system.^1^

Dose calculation algorithms in RTP systems can be broadly classified into measurement‐based and model‐based approaches. Measurement‐based models, such as the Clarkson algorithm,^2^ compute dose based on measurements in water. These models usually correct the homogeneous water distributions to account for treatment aids, patient contours, and tissue inhomogeneities. Unlike measurement‐based approaches, model‐based approaches, such as the convolution/superposition algorithm,^3^ compute the dose in water or patient from physics principles. The dose calculation accounts for beam energy, treatment aids, the transport of primary and secondary radiation inside the patient, and the effects of tissue inhomogeneities on the dose distributions.

When commissioning RTP dose calculation algorithms, the goal is often to achieve agreement between calculated and measured doses within 1–2 % for open and wedge fields in water. While this is possible to achieve using both measurement‐based and model‐based algorithms in water phantoms, such an agreement is usually not possible for measurement‐based algorithms in phantoms with heterogeneities. This is due to the fact that measurement‐based models are able to account for the effect of tissue inhomogeneities on the primary radiation. However, correcting for the scatter radiation is difficult since it depends on field size, beam energy and shape, location and density of the inhomogeneities.^4^ In contrast, model‐based algorithms can account for the effect of tissue inhomogeneities on the scatter radiation using the density scaling method^5^ ^,^ ^6^ or other approaches.^7^ ^,^ ^8^

Many RTP research and commercial systems still use measurement‐based models for dose calculations. The accuracy of dose calculation algorithms in heterogeneous phantoms has previously been investigated by comparing calculations with measurements. Comparisons were mainly limited to the central‐axis due to the complexity of performing accurate full three‐dimensional (3D) measurements.^4^ ^,^ ^9^ ^,^ ^10^ Therefore, there is a need for a detailed study and examination of 3D dose distributions in heterogeneous phantoms, since many studies have debated the use of inhomogeneity corrections in treatment planning, especially for lung treatments.^11^ ^–^ ^14^ In this work, we compared FOCUS RTP system dose distributions from the measurement‐based Clarkson^2^ and model‐based MultiGrid Superposition^15^ (MGS) algorithms with those from the beam ^16^ Monte Carlo (MC) code system in a set of heterogeneous phantoms. The phantom and beam geometries simulate clinical treatment situations. We present results that suggest turning‐off the effective path length inhomogeneity correction in the Clarkson model for lung treatments. While the methods presented are specific to the FOCUS Clarkson and FOCUS MultiGrid Superposition models, results should apply to other implementations of the Clarkson and Superposition models in other treatment planning systems.

## METHODS

The Clarkson and MGS models of the FOCUS RTP system are used (version 2.5.0, Computerized Medical Systems, St. Louis, MO). Calculated dose distributions are compared to beam Monte Carlo dose distributions for a Varian Clinac 2300C/D accelerator (Varian Oncology Systems, Palo Alto, CA). Dose distributions are normalized to the value from the beam simulation, at the depth of maximum dose $\left(d_{\text{max}}\right)$ along the central axis. Distributions for 6‐ and 15‐MV beams are compared for $5\times 5{\text{cm}}^{2}$ and $10\times 10{\text{cm}}^{2}$ fields.

In order to compare the beam and RTP dose calculation models, corresponding dose values are extracted from the 3D dose data from each model. The dose values from the corresponding planes are read into a comparison utility. The utility compares both sets of plane data and produces the percent‐difference relative to the dose at $d_{\text{max}}$, distance to agreement (distance to agreement to the nearest point exhibiting the same dose level) and percent passing information. The output is then read by another utility which allows the visualization of isodose distributions and various information.

### Heterogeneous phantoms

**Figure 1:**
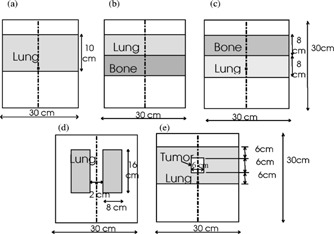
Schematic of the (a) lung slab phantom, (b) lung‐bone slab phantom, (c) bone‐lung slab phantom, (d) mediastinum phantom and (e) tumor phantom.

The five heterogeneous phantom geometries studied in this work are shown in Fig. 1. All phantoms have external dimensions of $30\times 30\times 30{\text{cm}}^{3}$. A build‐up layer of water with a thickness of 3 cm for the 6‐MV beam and 5 cm for the 15‐MV beam is used in order to achieve electronic equilibrium before entering the low or high density layers. The heterogeneities are assumed to have the same atomic composition as water.

The lung and bone slab geometries have been used to verify dose calculation algorithms along the central‐axis in many research articles.^4^ ^,^ ^9^ ^,^ ^10^ The slab‐based phantoms are used to examine doses inside the inhomogeneities, beyond them, and near material interfaces. Figure 1(a) is a lung slab in a water phantom with a density of $0.31g/{\text{cm}}^{3}$. The lung slab has a dimension of $30\times 30\times 10{\text{cm}}^{3}$. Figures 1(b) and 1(c) are a lung‐bone and bone‐lung slab in a water phantom with 0.2 and $2.4g/{\text{cm}}^{3}$ density for lung and bone, respectively. The lung and bone slabs have a dimension of $30\times 30\times 8{\text{cm}}^{3}$ each. To achieve full scatter conditions, the lateral extent of the slabs is larger than the $5\times 5{\text{cm}}^{2}$ and $10\times 10{\text{cm}}^{2}$ field sizes used in this work.

Figure 1(d) is a mediastinum geometry (two lung geometry).^17^ Each lung dimension is $8\times 30\times 16{\text{cm}}^{3}$ and has a density of $0.31g/{\text{cm}}^{3}$; the two lungs are separated by water of 2 cm thickness. For this geometry, the dose will change inside in the lungs. The scatter dose contribution to the central axis will also change without affecting the primary dose. The tumor geometry is shown in Figure 1(e) with a tumor density of $1g/{\text{cm}}^{3}$ and a $6\times 6\times 6{\text{cm}}^{3}$ dimension centered in a lung thickness of 18 cm. The tumor geometry is designed to simulate the treatment of a patient lung tumor, which is often located inside the lung.^4^ The setup is used to examine the dose distributions inside the tumor, lung and near interfaces.

### BEAM setup

beam is a Monte Carlo based system for modeling radiotherapy treatment unit heads for 3D RTP.^16^ The beam code is developed based on the EGS4 code system^18^ which has been demonstrated to accurately simulate the coupled transport of electrons and photons in matter.^19^ ^,^ ^20^ ^,^ ^22^ The EGS4 system has been used to calculate beam data for many clinical linear accelerators and dose distributions in both homogeneous and heterogeneous phantoms.^19^

The beam code generated the full phase‐space of all particles for the 6‐ and 15‐MV beams that emerge from the simulated Varian Clinac 2300C/D treatment unit. The phase‐space file contains information about particle type, energy, position, direction, weight, and a tag that records the particle history at any specified plane in the simulated geometry.

To commission the Monte Carlo simulated phase‐space data, the dose distributions calculated using the Monte Carlo method were compared with the measured beam data for the 6‐ and 15‐MV beams. The incident beam parameters, which include the incident electron energy, the spatial and the angular distributions at the target surface, were altered so that the calculated dose distributions can match the measured values within 2% of the maximum dose everywhere in the phantom.^22^ ^,^ ^24^ ^,^ ^25^ Sufficient number of particle histories were simulated to ensure the 1‐sigma statistical uncertainty on the calculated dose values was less than 1% of the maximum dose for all voxels.

dosxyz in the beam/EGS4 system is a code for simulating electron and photon transport in a Cartesian volume and scoring the energy deposition in the designated voxels. The geometry is a rectilinear volume with voxel dimensions which are variable in all three dimensions. Each voxel can have different materials with varying densities for use with CT data. dosxyz was used to calculate the dose in the heterogeneous phantoms, depicted in Fig. 1, on 0.5×0.5×0.5−cm grid size using the 6‐ and 15‐MV beam phase‐space source distributions.

### Dose calculation algorithms

The Clarkson sector integration algorithm uses patient data, treatment machine data, and setup information to simulate dose distributions inside the patient. The patient information consists of relative electron density data which represents a section of the patient. The relative electron density values can be calculated from either the CT data or the assigned structure densities. The algorithm takes into account primary dose corrections for inhomogeneities in the patient, transmission by the wedge, and scatter modifications of blocks and collimators resulting from field shaping. The algorithm does not take into account scatter modifications due to differences in field intensity (e.g., wedges), patient density, surface curvature and missing tissue.

In the Clarkson algorithm, the dose is calculated at a point (*x,y*) in a plane at depth *d* as the sum of primary and scatter dose:

(1) $$D(x,y,d)=\Phi (x,y)\left\lfloor \text{TAR}(0,d_{eff})+\text{SAR}(x,y,d_{eff})\right\rfloor .$$

TAR is the central‐axis tissue‐to‐air ratio at the radiological depth $d_{\max}$ extrapolated to zero field size. SAR is the scatter‐to‐air ratio at $d_{\max}$ at the dose point. Here, the Clarkson algorithm accounts for the effects of tissue inhomogeneities by calculating the primary and scatter dose along each beam “effective path length” fan line. Note that the Clarkson algorithm, unlike the MGS algorithm, does not model the effect of tissue inhomogeneities on scatter dose distribution.

The MGS algorithm uses fundamental physics principles to calculate dose distributions inside the patient rather than providing reproductions or modifications of measured data. Specifically, the MGS algorithm computes the dose by convolving the total energy released in the patient with Monte Carlo generated energy deposition kernels.^20^ The MGS model accounts for beam hardening, missing tissue and the effects of tissue inhomogeneities on the dose distributions. A detailed description of the implementation and commissioning of the MGS model has recently been reported.^15^ In the MGS algorithm, the total energy released per unit mass (TERMA^21^), *T*, at the interaction point may be calculated as

(2) $$T({\underline{r}}^{\prime },E)=\frac{\mu }{\rho }(E,\underline{r^{\prime }})E\Phi ({\underline{r}}_{o})\exp(-\mu |r^{\prime }-r_{o}|),$$

where *μ/ρ* is the mass attenuation coefficient, *E* is the beam energy, *μ* is the linear attenuation coefficient, ${\underline{r}}_{o}$ is a point at the patient surface, Φ is the primary fluence along the beam fan line through the position ${\underline{r}}_{o}$. The dose at a point $\underline{r}$ is calculated by convolving TERMA with a density scaled energy deposition kernel,

(3) $$D(\underline{r})=\int \int T({\underline{r}}^{\prime },E)\frac{\rho ({\underline{r}}^{\prime })}{\bar{\rho }}K(\bar{\rho }l(\underline{r}-{\underline{r}}^{\prime }),\underline{r}-{\underline{r}}^{\prime },E)d^{3}r^{\prime }dE,$$

where *K* is the energy deposition kernel, $\bar{\rho }$ is the average density along the path between the interaction and dose deposition sites, $l(\underline{r}-{\underline{r}}^{\prime }),\underline{r}-{\underline{r}}^{\prime }$ is the distance between interaction and dose deposition sites, and $\rho ({\underline{r}}^{\prime })$ is the density of the interaction site. *K* is distorted by the radiological distance $\bar{\rho }l(\underline{r}-{\underline{r}}^{\prime })$ from dose deposition point. The density scaling method was used to scale the energy deposition kernels to account for the effect of tissue inhomogeneities on the dose distribution.

## RESULTS AND DISCUSSION

**Figure 2:**
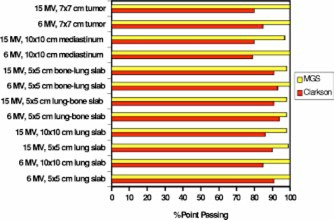
(Color) A histogram showing the total number of Clarkson and MGS dose calculation points passing a 3% or 3 mm accuracy criteria versus beam.

Figure 2 shows the total number of points that passed a 3% or 3 mm accuracy criteria for the Clarkson and MGS models, compared to beam, for all study beam setups and test phantoms. This is an overall quantitative assessment of the accuracy through reporting the total number of calculation points passing a 3% or 3 mm criteria. In general, the MGS model is more accurate than the Clarkson model in all phantoms with an average of 99% versus 87% for the Clarkson model.

Next, we will discuss the results of the quantitative evaluation for a qualitative evaluation. Specifically, the depth dose and isodose distributions in each test phantom were examined individually to study the effects of tissue inhomogeneities on dose distributions and to determine the accuracy of the algorithms under these various clinical situations. For the sake of brevity, we decided to present only the MGS model isodose results.

### Lung slab phantom

**Figure 3:**
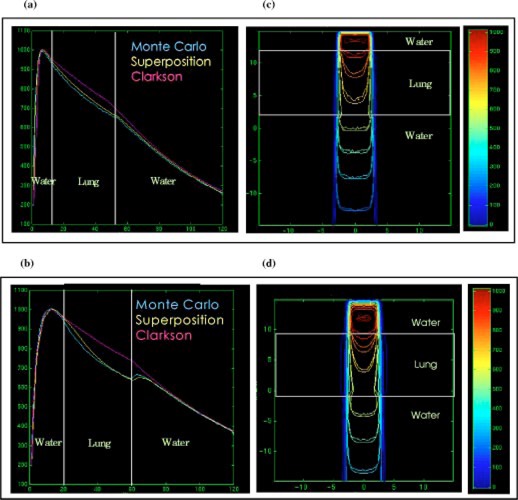
(Color) Depth dose distributions for the Clarkson, MGS, and beam in the lung phantom, (a) $5\times 5{\text{cm}}^{2}$, 6‐MV beam, (b) $5\times 5{\text{cm}}^{2}$, 15‐MV beam. Isodose distributions for the MGS and beam along a central‐axis transverse plane, (c) $5\times 5{\text{cm}}^{2}$, 6‐MV beam, (d) $5\times 5{\text{cm}}^{2}$, 15‐MV beam.

**Figure 4:**
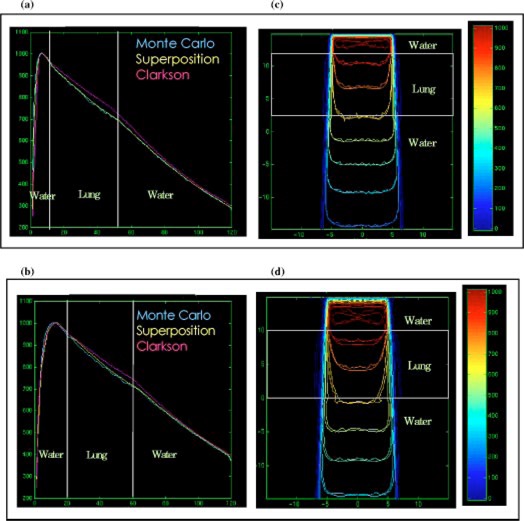
(Color) Depth dose distributions for the Clarkson, MGS, and beam in the lung phantom, (a) $10\times 10{\text{cm}}^{2}$, 6‐MV beam, (b) $10\times 10{\text{cm}}^{2}$, 15‐MV beam. Isodose distributions for the MGS and beam along a central‐axis transverse plane, (c) $10\times 10{\text{cm}}^{2}$, 6‐MV beam, (d) $10\times 10{\text{cm}}^{2}$, 15‐MV beam.

Figures 3(a) and 3(b) show the Clarkson, MGS, and beam depth dose distributions and Figs. 3(c) and 3(d) show the MGS and beam isodose distributions in the lung phantom for the $5\times 5{\text{cm}}^{2},6-$ and 15‐MV beams. Figures 4(a)–4(d) show the same information for the $10\times 10{\text{cm}}^{2},6-$ and 15‐MV beams. Note that the beam MC dose distributions are not smooth as a consequence of the inherent random errors, or statistical uncertainty, contained in the calculations.^23^

For this type of setup, the MGS results are within 3% or 3 mm of beam results. Note however, that the Clarkson model overestimates the dose by a maximum of 10% and 4%, compared to beam, in the lung for the $5\times 5{\text{cm}}^{2}$ and $10\times 10{\text{cm}}^{2}$ field sizes, respectively. The overestimation arises from the use of the effective path length correction which tends to boost the primary dose in the low density region without accounting for loss of scatter.

In reality, a deficit in dose in the lungs occurs, mainly due to two conditions presented inside the lungs: (i) the loss of scatter and (ii) electronic disequlibrium, especially for higher energies and small field sizes as shown in Fig. 3. This is due to the increase of the ratio of primary to scatter radiation. With higher energy beams, there is a reduction in the number of scattered photons. Further, more energy is carried away by primary electrons from the low density lung region than re‐enters from the off‐axis direction. The increase of primary photons in lung also results in an increase (build‐up) in the dose when radiation penetrates through the water region below the lung slab as shown in Fig. 3(b). The effect of lung inhomogeneity along central‐axis dose distribution increases with smaller field sizes and higher energies.

The MGS model can account for most of these effects by scaling the energy deposition kernels using the density scaling method as we observed in the figures. The Clarkson model, using only the effective path length correction for the primary radiation, does not account for these scatter effects and therefore yields inaccurate results.

The protuberance of the 3% and 5% isodose lines in lung, as shown in Figs. 3(c) and 3(d), is modeled correctly using the MGS algorithm. The bulge of low isodose lines is due to the fact that higher energy photons set primary electrons in motion with higher energy resulting in an increased electron range, especially in low density materials. The increase in the lateral range of electrons and scattered photons in low density regions causes an increase in the lateral range of dose. This usually results in penumbra broadening in low density regions such as lung, especially for small field sizes and higher energy beams.

### Lung‐bone and bone‐lung slab phantoms

**Figure 5:**
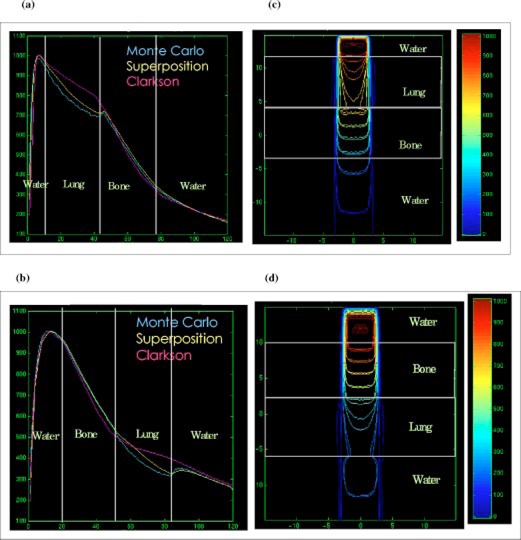
(Color) Depth dose distributions of the Clarkson, MGS, and beam for $5\times 5{\text{cm}}^{2}$, 15‐MV beam in the (a) lung‐bone phantom, (b) bone‐lung phantom. Isodose distributions for the MGS and beam along a central‐axis transverse plane for $5\times 5{\text{cm}}^{2}$, 15‐MV beam in the (c) lung‐bone phantom, (d) bone‐lung phantom.

Figures 5(a) and 5(c) show the depth dose distribution and isodose lines for $5\times 5{\text{cm}}^{2}$, 15‐MV beams in the lung‐bone slab phantom. Figures 5(b) and 5(d) show the dose distributions for the same beam setup in the bone‐lung slab phantom.

In these phantoms, the MGS model is more accurate than Clarkson in the lung and bone structures. The increased accuracy for the MGS model is due to the correct modeling of the inhomogeneities effects on scatter radiation in the MGS algorithm, using the density scaling method. In the bony structure, both models generate results within 3% of BEAM. The reason for this agreement is that most electrons set in motion by photons deposit the energy locally due to the high density of bone which limits electron range. Note that the Clarkson model, MGS model, and beam assume that the bone material is equivalent to water (i.e., same atomic properties) in the dose calculations. Larger differences between the MGS and beam would be expected if we had modeled the materials as nonwater equivalent in the beam calculations, especially near bone interfaces.

The differences observed in the lung slab phantom, using the Clarkson model, in the build‐down and build‐up regions are also observed in the lung‐bone and bone‐lung phantoms. There are no interface effects after the radiation penetrates the water below the bone in the lung‐bone phantom. The reason for this behavior is the reduction of both primary and scatter radiation in bone due to attenuation.

### Mediastinum phantom

**Figure 6:**
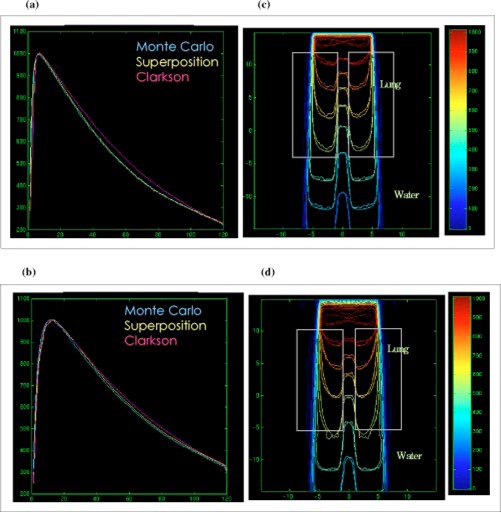
(Color) Depth dose distributions for the Clarkson, MGS, and beam in the mediastinum phantom, (a) $10\times 10{\text{cm}}^{2}$, 6‐MV beam, (b) $10\times 10{\text{cm}}^{2}$, 15‐MV beam. Isodose distributions for the MGS and beam along a central‐axis transverse plane, (c) $10\times 10{\text{cm}}^{2}$, 6‐MV beam, (d) $10\times 10{\text{cm}}^{2}$, 15‐MV beam.

Figures 6(a)–6(d) show the depth dose and isodose lines in the two‐lung geometry for $10\times 10{\text{cm}}^{2}$ 6‐MV and 15‐MV beams. The phantom shape alters the scatter contribution to the central‐axis without modifying the primary radiation along the central axis. Therefore, comparing dose calculation models along the central axis only for such type of phantom may lead to incorrect conclusions on the accuracy of models.

The MGS model agrees with beam along the central‐axis and the Clarkson model overestimates the dose. The dose distributions inside the lung and at the lateral interfaces show that the MGS model predicts the penumbra broadening inside the lung and the build‐down and build‐up along the interfaces, especially for the 15‐MV beam.

### Tumor phantom

**Figure 7:**
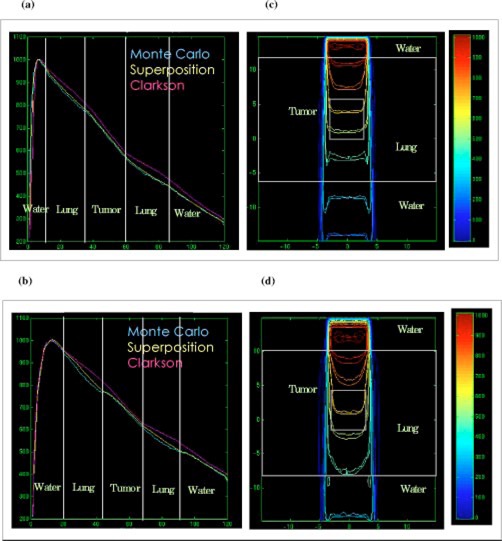
(Color) Depth dose distributions for the Clarkson, MGS, and beam in the tumor phantom, (a) $7\times 7{\text{cm}}^{2}$, 6‐MV beam, (b) $7\times 7{\text{cm}}^{2}$, 15‐MV beam. Isodose distributions for the MGS and beam along a central‐axis transverse plane, (c) $7\times 7{\text{cm}}^{2}$, 6‐MV beam, (d) $7\times 7{\text{cm}}^{2}$, 15‐MV beam.

The tumor geometry shown in Figures 7(a)–7(d) depicts a clinical setting of treating a tumor inside the lung using a $7\times 7{\text{cm}}^{2}$ field. The MGS model is more accurate than the Clarkson along the central axis. The results show the MGS model is within 2% for 6 MV and 3% for 15 MV, compared to beam. Further, the isodose distributions in Figures 7(c) and 7(d) show that the MGS predicts the doses correctly along and beyond the inhomogeneity in the lateral direction. In contrast, the Clarkson model overestimates the dose in the lung by a maximum of 6% in the tumor and by a maximum of 10% in lung for the 15‐MV beam.

These results clearly show that using the Clarkson model (with only the effective path length correction) overestimates the dose in lung and target. This may lead the “treatment planner” to reduce the dose in order to reduce lung complications. Consequently, this may result in underdosing the target. Therefore, inhomogeneity correction, based only on the effective path length correction, should not be used for Clarkson‐type algorithms in lung treatments because it may lead to inaccurate calculations. The results and analysis presented so far suggest treating the patient as a unit density medium in order to avoid underdosing the target. Furthermore, the results indicate that using model‐based algorithms for heterogeneous calculations, such as the MGS model, is necessary to achieve accurate dose distributions in lung treatments.

## CONCLUSIONS

Accurate dose calculation models in RTP systems are a vital link in the radiotherapy treatment planning process. We compared dose distributions from the FOCUS Clarkson and FOCUS MultiGrid Superposition (MGS) models with those from the beam Monte Carlo simulation system in heterogeneous phantoms. The results confirmed that the MGS model and beam doses are within 3% or 3 mm, and the MGS model is more accurate than the Clarkson model in heterogeneous phantoms. Furthermore, the results suggest turning‐off the effective path length inhomogeneity correction for lung treatments using the measurement‐based Clarkson model and demonstrate the importance of using the model‐based MGS algorithm for heterogeneous dose calculations.
